# Recovery of Barotrauma Injuries in Chinook Salmon, *Oncorhynchus tshawytscha* from Exposure to Pile Driving Sound

**DOI:** 10.1371/journal.pone.0039593

**Published:** 2012-06-22

**Authors:** Brandon M. Casper, Arthur N. Popper, Frazer Matthews, Thomas J. Carlson, Michele B. Halvorsen

**Affiliations:** 1 Department of Biology, Center for Comparative and Evolutionary Biology of Hearing University of Maryland, College Park, Maryland, United States of America; 2 Pacific National Northwest Laboratory, Marine Sciences Laboratory, Sequim, Washington, United States of America; Texas A&M University-Corpus Christi, United States of America

## Abstract

Juvenile Chinook salmon, *Oncorhynchus tshawytscha*, were exposed to simulated high intensity pile driving signals to evaluate their ability to recover from barotrauma injuries. Fish were exposed to one of two cumulative sound exposure levels for 960 pile strikes (217 or 210 dB re 1 µPa^2^·s SEL_cum_; single strike sound exposure levels of 187 or 180 dB re 1 µPa^2^⋅s SEL_ss_ respectively). This was followed by an immediate assessment of injuries, or assessment 2, 5, or 10 days post-exposure. There were no observed mortalities from the pile driving sound exposure. Fish exposed to 217 dB re 1 µPa^2^·s SEL_cum_ displayed evidence of healing from injuries as post-exposure time increased. Fish exposed to 210 dB re 1 µPa^2^·s SEL_cum_ sustained minimal injuries that were not significantly different from control fish at days 0, 2, and 10. The exposure to 210 dB re 1 µPa^2^·s SEL_cum_ replicated the findings in a previous study that defined this level as the threshold for onset of injury. Furthermore, these data support the hypothesis that one or two *Mild* injuries resulting from pile driving exposure are unlikely to affect the survival of the exposed animals, at least in a laboratory environment.

## Introduction

Anthropogenic noise in aquatic environments has developed into an issue of worldwide concern due to its potential negative effects on animal life. This concern has been well documented for marine mammals [1], [2], [3], [4], but has only recently become prominent as a potential issue for fishes [4], [5], [6], [7], [8], [9], [10], [11]. Of particular interest and concern are the potential effects of the intense sounds produced by in-water pile driving used for construction and repair of bridges and infrastructure, as well as offshore wind farms.

Despite the concern that pile driving could harm fishes, it was only during construction of the east span of the San Francisco Bay Bridge in California in 2008 that federal and state regulators on the US West Coast established interim criteria for onset of tissue damage to fishes that might occur as a result of exposure to pile driving [12], [13]. These interim criteria were based upon the total amount of sound to which fishes were exposed during a pile driving operation, and were addressed as accumulated sound exposure levels (SEL_cum_). The interim SEL_cum_ was set at 187 dB re 1 µPa^2^·s for fishes greater than 2 g [12], [13]. This interim level was based on very little quantitative data on pile driving effects [10] and thus has been a subject of debate [9], [10]. At the same time, having only limited data upon which to set criteria, these interim criteria were intentionally conservative in consideration of the ESA (US Endangered Species Act) listed fishes likely to be exposed [9], [10]. Scientists and regulators involved in the regulatory rulings expected that future research would produce better data for the response of fishes to pile driving sound exposures as well as a more thorough understanding of the effects of pile driving on fish physiology [14], [15].

The data needed to set criteria is based on the effects on fish from pile driving exposure measured as either physical damage to tissues and organs (i.e. barotrauma) [16], [17], and/or behavioral changes, such as avoidance of important spawning or nursery areas, migratory pattern alteration, or vacating from biologically important locations. There are, however, few reliable data regarding the potential impacts on fish species when exposed to pile driving [9], [10], except for two recent barotrauma injury studies [16], [18].

The lack of data on the effects of pile driving sound (or other intense sounds) on fishes results from the difficulty in performing controlled studies with fishes near pile driving activities [9]. Factors limiting such studies have included safety of investigators as well as logistical issues, including the inability of investigators to get permission to control the parameters of expensive commercial pile driving projects. Furthermore, maintenance of fishes in appropriate condition for a study (i.e., neutrally buoyant state [16], [17]), is also difficult to control in field studies.

A recent study by Halvorsen et al. [16] overcame the aforementioned limitations and examined the effects of pile driving on juvenile Chinook salmon, *Oncorhynchus tshawytscha*. This species was chosen for study since specific populations are currently listed on the ESA as threatened and/or endangered on the US West Coast due to many anthropogenic factors including exposure to pile driving and other impulsive sources.

Halvorsen et al. [16] developed a High Intensity Controlled Impedance Fluid-filled wave Tube (HICI-FT) that could replicate actual pile driving sounds, including the number of impulsive strikes, the signal spectra, and sound level. Using the HICI-FT, it is also possible to present the impulsive signals under far-field, plane wave acoustic conditions, thereby providing a controlled exposure paradigm that is not easily achievable in a field setting. In the laboratory, investigators were able to monitor the buoyancy state of the fish and provide the opportunity for fish to become neutrally buoyant just before treatments began. After exposures, barotrauma injuries were immediately assessed to define an injury onset threshold from pile driving exposure.

The Halvorsen et al. [16] study demonstrated that a cumulative SEL (SEL_cum_) of 210 dB re 1 µPa^2^⋅s was derived from a single strike SEL (SEL_ss_) of 180 dB re 1 µPa^2^⋅s SEL_ss_ for 960 pile strikes, and from SEL_ss_ of 177 dB re 1 µPa^2^·s for 1920 pile strikes are the metrics that best define the threshold for onset of injury. These results provided the first direct evidence that the current industry regulations are too conservative relative to levels in which actual injury occurs in juvenile Chinook salmon.

While establishing the threshold for injury onset [16], an important question arose as to whether fish would be able to recover from barotrauma injuries, or if some of these injuries would result in delayed mortality. Thus, a study of delayed mortality (or recovery) would provide insight into whether exposure to pile driving sounds could result in delayed onset injuries. With this is mind, the current study examined the recovery of juvenile Chinook salmon resulting from injuries sustained at two different SEL_cum_ levels of pile driving. Evaluation of recovery was accomplished by sampling at four time points following exposure to document injuries and injury recovery response.

## Materials and Methods

### Ethics Statement

Experiments were conducted under supervision and approval of the Institutional Animal Care and Use Committee (IACUC) of the University of Maryland (protocol #R-09-23). Fish were held under authority of the Maryland Department of Natural Resources (Natural Resources Articles 4-602 and 4-11A-02).

### Study Fish

Juvenile Chinook salmon, (99.4±8.49 mm SL and 10.1±3.24 g) obtained from Pacific Northwest National Laboratory from the Priest Rapids Hatchery in Mattawa, Washington, were used in this study. After arrival at the University of Maryland, via overnight air delivery, the fish were acclimated for a minimum of two weeks before being used in experiments. They were maintained on a 14:10 light/dark cycle in 235 gallon round tanks at 14°C in recirculated filtered fresh water. Fish were fed three times per week except during the week of exposure in which they were not fed. All fish were tail clipped to distinguish individuals.

### Pile Driving Exposure Equipment and Signal Presentation

Pile driving exposure was conducted using the HICI-FT that had a stainless steel chamber 45 cm long with a 25 cm internal diameter and 3.81-cm-thick. Large shakers on either end of the chamber were used to create sounds that accurately reproduced the acoustic characteristics and sound levels of pile driving sounds under far-field plane wave acoustic conditions. For a detailed description of the equipment and development see Halvorsen et al. [16].

Signal generation and data acquisition for the HICI-FT are described in detail in Halvorsen et al. [16]. In brief, pile driving sounds used in this study were field recordings taken at a range of 10 m from a 76.2 cm steel shell pile (outer diameter) driven using a diesel hammer at the Eagle Harbor Maintenance Facility [19]. Eight different recordings of individual pile driving strikes were used in the exposures and were normalized to the same SEL and compiled into a single file that contained 12 repetitions of each of the 8 strikes, for a total of 96 strikes. These 96 strikes were randomized each day using MATLAB (The MathWorks, Inc., Natick, Massachusetts). The randomized sequence of sounds was presented to fish and repeated 10 times for a 960-strike presentation. Therefore, each day’s fish received a different presentation of pile strikes.

### Fish Exposure

The HICI-FT has an acrylic acclimation chamber (0.062 m^3^) mounted around the opening of the exposure chamber (0.022 m^3^) and both were filled with filtered, dechlorinated water. Four fish were released into the acclimation chamber and allowed to swim freely, with the entrance to the exposure chamber blocked, for a 20 minute acclimation period. After 20 minutes, the buoyancy state was documented and the fish were allowed into the exposure chamber and the upper shaker/lid was sealed over the chamber opening. The acclimation chamber was drained and the HICI-FT was rotated from the vertical position to the horizontal position for each exposure or control treatment. When the exposure or control was completed, fish were removed from the chamber. One fish was immediately necropsied for barotrauma assessment and the other three were returned to tanks for recovery periods of 2, 5, or 10 days for post-exposure assessment. Those three fish’s feeding and swimming behaviors were observed and at 2, 5, or 10 days post-exposure fish were randomly selected for necropsy. Feeding behavior was documented by noting which fish were eating food pellets at all feeding periods, while swimming behavior was documented as swimming throughout the tank in a manner similar to behavior prior to exposure rather than sitting on the bottom or obvious labored swimming movements.

Two exposure paradigms were used for this study: Exposure 1 presented 960 strikes at levels of 217 dB re 1 µPa^2^·s SEL_cum_, using 187 dB re 1 µPa^2^·s SEL_ss_; Exposure 2 presented 960 strikes at levels of 210 dB re 1 µPa^2^·s SEL_cum_ using180 dB re 1 µPa^2^·s SEL_ss_. As the equal energy hypothesis was demonstrated to be false by Halvorsen et al [16] they reported that SEL_cum_ alone is not sufficient to predict the risk of injury to fish exposed to impulsive sound, therefore it is important that all three metrics are reported together. From here forward, the study will refer to Exposure 1 or Exposure 2 to simplify the reference to the exposure paradigms.

A total of 175 fish were exposed and 53 were used as controls and subject to the identical process as exposed fish but without the pile driving sound. The two exposure parameters were selected because the lower level (Exposure 2) was at the threshold for physical injury identified by Halvorsen et al. [16] and the higher level (Exposure 1) presented about four times as much energy.

All necropsies were conducted “blind,” so that the investigator performing the dissections had no knowledge of whether each fish was an exposed sample or a control. For necropsy, fish were euthanized in a buffered MS-222 solution and examined for external signs of barotrauma (e.g. damage to eyes, fins, gills) utilizing methodology from previous Chinook salmon [16], [17]. Each potential injury was noted as present or not (for a detailed list of all potential external and internal barotraumas injuries see Halvorsen et al. [16]. Following the external assessment, fish were assessed internally. After the more ventral internal organs (e.g. stomach, intestines) were examined for injury they were carefully moved aside to examine deeper organs and tissues (e.g., swim bladder and kidney).

### Evaluation of Barotrauma Injuries and Recovery

In the previous study [16], an injury evaluation index (Injury Index) was developed based on assessment of the physiological significance of the range of barotrauma injuries observed. The injury classification of *Mild*, *Moderate*, or *Mortal* were also used in this study.

The injury evaluation index weighted all observed injuries equally because some injuries (i.e. many of the hematomas) appeared at higher frequencies during the later post-exposure days. Furthermore, the probability of detection of injuries was inconsistent with the probability of the occurrence of these injuries. The injury index was calculated by determining the ratio of frequency of occurrence of injuries for each individual injury at each time point and multiplied by 100, and then summed together.
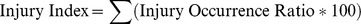



### Statistical Analysis

Two-way ANOVA tests with Bonferroni correction on the multiple comparisons (SigmaPlot 11, SYSTAT Software, Inc.) were used to evaluate any differences between Exposure 1 and 2 and post exposure days in terms of both injury index values as well as number of injuries observed. All statistical information is displayed in Table 1.

**Table 1:** Summary of statistical analyses.

Variables Being Compared	Test	F Value	*p* Value
Number of injuries observed between Exposure 1 and Exposure 2	ANOVA	F_1, 167_ = 10.129	*p*<0.001
Injury Index Value between Exposure 1 and Exposure 2	ANOVA	F_1, 3_ = 17.466	*p = *0.025
Number of injuries observed on Day 0 post exposure between Exposure 1 and Exposure 2	ANOVA	F_3, 167_ = 7.650	*p*<0.001
Number of injuries observed on Day 2 post exposure between Exposure 1 and Exposure 2	ANOVA	F_3, 167_ = 6.862	*p*<0.001
Number of injuries observed on Day 5 post exposure between Exposure 1 and Exposure 2	ANOVA	F_3, 167_ = 2.621	*p = *0.045
Number of injuries observed on Day 10 post exposure between Exposure 1 and Exposure 2	ANOVA	F_3, 167_ = 3.271	*p = *0.036
Number of injuries observed between Day 0 and Day 2 post exposure within Exposure 1	ANOVA	F_3, 167_ = 0.404	*p>*0.05
Number of injuries observed between Day 0 and Day 5 post exposure within Exposure 1	ANOVA	F_3, 167_ = 3.704	*p = *0.008
Number of injuries observed between Day 0 and Day 10 post exposure within Exposure 1	ANOVA	F_3, 167_ = 5.035	*p*<0.001
Number of injuries observed between Day 2 and Day 5 post exposure within Exposure 1	ANOVA	F_3, 167_ = 3.443	*p = *0.040
Number of injuries observed between Day 2 and Day 10 post exposure within Exposure 1	ANOVA	F_3, 167_ = 4.424	*p*<0.001
Number of injuries observed between Day 5 and Day 10 post exposure within Exposure 1	ANOVA	F_3, 167_ = 1.348	*p>*0.05
Number of injuries observed between Day 0 and Day 2 post exposure within Exposure 2	ANOVA	F_3, 167_ = 0.115	*p>*0.05
Number of injuries observed between Day 0 and Day 5 post exposure within Exposure 2	ANOVA	F_3, 167_ = 1.056	*p>*0.05
Number of injuries observed between Day 0 and Day 10 post exposure within Exposure 2	ANOVA	F_3, 167_ = 1.023	*p>*0.05
Number of injuries observed between Day 2 and Day 5 post exposure within Exposure 2	ANOVA	F_3, 167_ = 1.145	*p>*0.05
Number of injuries observed between Day 2 and Day 10 post exposure within Exposure 2	ANOVA	F_3, 167_ = 0.891	*p>*0.05
Number of injuries observed between Day 5 and Day 10 post exposure within Exposure 2	ANOVA	F_3, 167_ = 2.013	*p>*0.05

## Results

**Figure 1:**
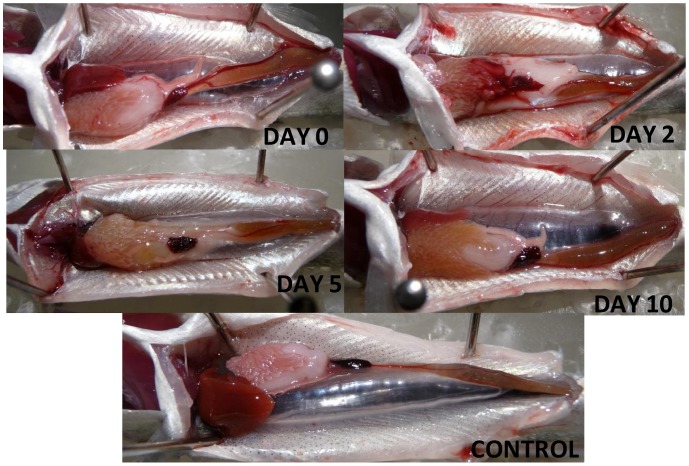
Photos of example injuries. Ventral view of Chinook salmon, for Exposure 1 with recovery periods of 0, 2, 5, and 10 days post-exposure as well as an example of a control fish. Day 0 displays hematomas of the swim bladder, liver, and adipose tissue, as well as hemorrhaging of the intestine. Day 2 displays hemorrhaging of the spleen and hematomas of the intestine and adipose tissue. Day 5 displays a hematoma of the intestine. Day 10 displays a fish with no visible injuries, though mottling of the spleen (raspberry appearance) can be observed which was present in most fish that were exposed to sound pile driving sounds, and not usually present in control fish. The anterior ends of all fish are orientated to the left.

**Figure 2:**
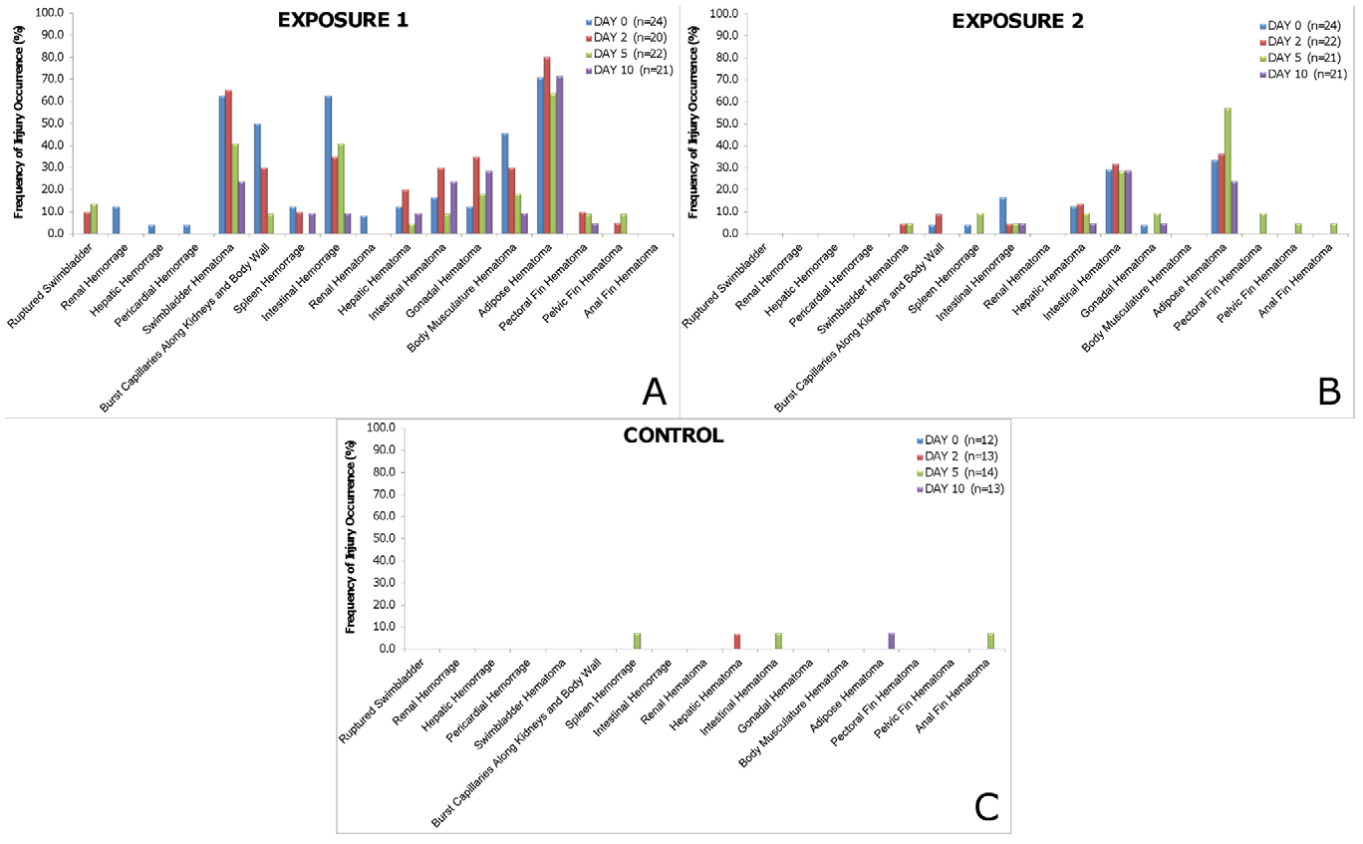
The frequency of occurrence of injuries from Exposure 1, Exposure 2, and the controls. Frequency of occurrence of injuries observed in Chinook salmon for Exposure 1(A), Exposure 2 (B), and control (C) at each of the four sample times post exposure. For a more detailed analysis of the individual injuries and their physiological significance please refer to Halvorsen et al. [16].

None of the fish in Exposure 1 or 2 died from barotrauma injuries out to 10 days post-exposure. Fish evaluated immediately (day 0) showed a wide range of injuries (Figure 1) that were similar to those reported by Halvorsen et al. [16]. Observed injuries most commonly included bruising of organs, while hemorrhaging of various tissues were observed much less frequently (Figure 2). It should be noted that even with the presence of these injuries, the sound exposed fish were still able to obtain and digest fish food pellets as well as display normal swimming behaviors post exposure.

**Figure 3:**
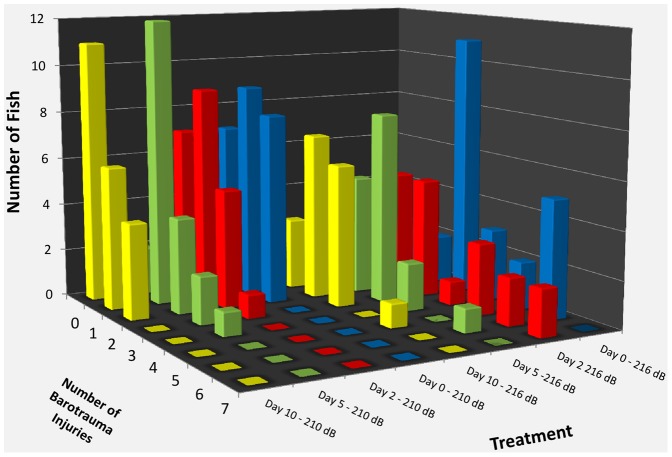
Number of observed injuries. Injuries that were observed in Chinook salmon for Exposure 1 and 2 as well as the different days post-exposure. There were a higher number of injuries observed at Exposure 1 versus Exposure 2. Numbers of injuries observed were higher at the earlier days post-exposure for Exposure 1 compared with the later days indicating that healing was occurring.

**Figure 4:**
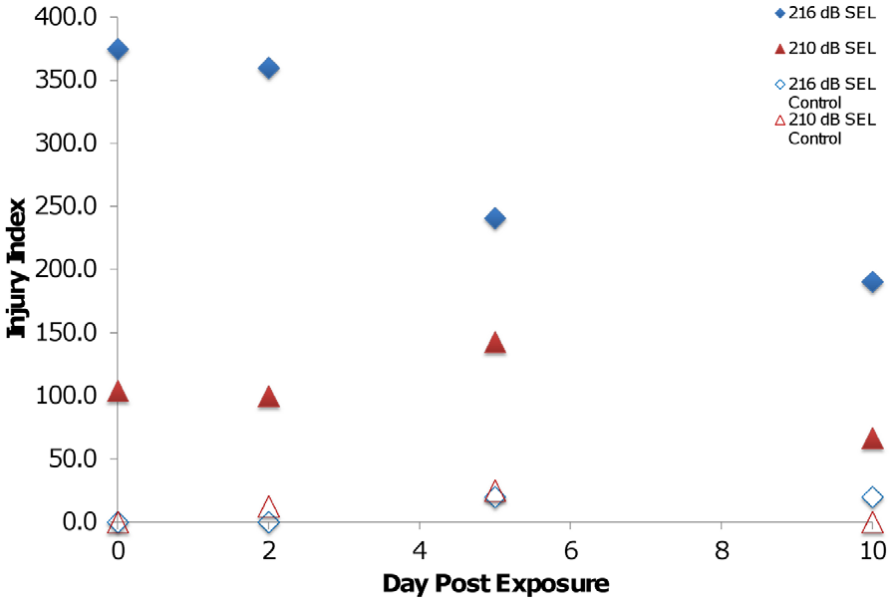
Injury index values for Chinook salmon. The injury index values for Exposure 1 and 2 to which Chinook salmon were exposed. There was a significant decrease in RWI scores from day 2 to day 5 in Exposure 1 fish (solid blue diamonds), suggesting that this is when healing is beginning to occur. There were no differences between days 5 and 10, implying that after initial healing of more serious injuries occurs, it then may take longer for minor injuries such as hematomas to heal. There was no significant difference between days 0 through 10 for fish in Exposure 2 (solid red triangles) and each day was not significantly different than controls (open blue diamond for Exposure 1 and open red triangle for Exposure 2).

A higher number of injuries were observed in fish in Exposure 1 than in Exposure 2 (Table 1) (Figure 3). Fish in Exposure 1 commonly exhibited swim bladder hematomas, burst capillaries, intestinal hemorrhages and hematomas, and hematomas of the gonads, adipose, and body musculature, while fish in Exposure 2 generally displayed only intestinal and adipose hematomas (Figure 2). As a result, fish in Exposure 1 had a significantly higher injury index value than fish from Exposure 2 (Table 1) (Figure 4). There were significant differences in the number of injuries observed per fish for each day post-exposure between Exposure 1 and 2 (Table 1) (Figure 3).

### Comparisons between Days within each Treatment

Chinook salmon in Exposure 1 showed higher frequencies of occurrence of injuries observed at day 0 and at day 2 post-exposure (Figure 3) (Table 1). There was, however, no significant difference between the number of injuries when comparing days 0 and 2 or when comparing days 5 and 10 (Table 1). By day 10 there was an average of only 1.90 injuries observed per fish. While there was no significant difference in the injury index values among the different days post-exposure (Table 1), visual examination of Figure 4 shows a drop of 120 injury index points between days 0 and 2 versus days 5 and 10, which implies that recovery of most injuries likely began after day 2. Recovery was further indicated by the decrease in the frequency of occurrence of each injury across the sample days as shown in Figure 2.

Fish subject to Exposure 2 averaged between 0.5 and 1.5 injuries per fish (Figure 3), and of the 81 fish tested 27 (33%) incurred no injuries. The number of injuries observed per fish did not differ significantly from control fish at any days post exposure (Table 1). The average injury index values at each day post-exposure were also low, with values on all days lower than those observed at any day in Exposure 1 fish (Figure 4).

## Discussion

This study followed upon previous research on pile driving sound effects on Chinook salmon [16], which established the onset of barotrauma injuries from pile driving as 210 dB re 1 µPa^2^·s SEL_cum_ derived from 180 dB re 1 µPa^2^·s SEL_ss_ with 960 impulsive signals. This current study independently replicated the onset level of barotrauma injury reported earlier and investigated the potential for injury recovery incurred from pile driving exposure up to 10 days post-exposure in a laboratory setting.

Chinook salmon in Exposure 1, showed a decrease in the number of injuries observed per fish (Figure 3) as well as the number of *Mortal* and *Moderate* injuries observed (Figure 2) suggesting some level of recovery in fish examined by days 5 and 10 post-exposure. This is further supported by observing a general trend (Figure 4) of decreasing injury index values from days 0 and 2 to days 5 and 10. The number of injuries observed were significantly higher at day 0 and 2 and there was a wide range of injuries observed from *Mild* to *Mortal* (Figure 2) based on the established injury classification system [16]. Days 0 and 2 were not statistically different from one another in terms of injury index, suggesting that the healing processes might begin after day 2. As the amount of time after exposure increased, the number of observed injuries significantly decreased from day 2 to day 5, as did the injury class of the remaining injuries. There was no significant difference in the number of injuries observed between days 5 and 10, leading to the suggestion that hematomas, the primary observed injuries at day 10, need more time to heal.

On average, Chinook salmon in Exposure 2 had fewer than 1.5 injuries per fish at any of the days post-exposure, and only *Moderate* and *Mild* injuries were observed. Furthermore, the number of injuries observed in exposed fish were not significantly different from control fish, and all four days had lower injury index values than those observed in Exposure 1 fish.

There were several instances of injuries appearing in samples at days 2 and 5 post-exposure that were not observed in the day 0 sample (Figure 2). These injuries include the presence of ruptured swim bladder, hepatic-, intestinal-, and gonadal- hematomas from Exposure 1while injuries from Exposure 2 included bruised swim bladder, burst capillaries, spleen hemorrhage, and gonadal hematomas. The explanation for the observation of injuries on day 5 that were not observed at day 0 or 2 is likely because the study was cross sectional and not longitudinal in design and fish were sacrificed to obtain the internal physical injury. In cross-sectional studies it is possible to observe responses that have an overall low probability of occurring and longer recovery time requirements during later post-exposure sampling periods. Such observations occur when a fish experiences a low probability injury at exposure but is randomly sampled for examination at a late post-exposure date.

### Interpretation of these Results

These results provide additional information to aid development of guidelines for the protection of aquatic animals from pile driving and other anthropogenic noise sources. The results from this study and its predecessor [16] show that juvenile Chinook salmon can be exposed to pile driving sounds substantially louder than the current industry guidelines of 187 dB re 1 µPa^2^·s SEL_cum_ [12], [13] and are either not injured or sustain injuries that are not fatal and appear to be recoverable in a laboratory setting. It should be acknowledged that these results are specifically for juvenile Chinook salmon and that it is possible that adult Chinook salmon and other species of salmonids could respond differently to these pile driving stimuli [15].

This study was conducted in a laboratory environment in which sound exposure levels and numbers of pile strikes were controlled. The recovery encountered in this study does not necessarily mean that fish in the wild would recover in the same manner since fish in the wild have to deal with numerous factors (e.g., having to seek food and avoid predators) that are not encountered in the lab. At the same time, it is likely that the level and duration of exposure of the lab animals was substantially greater than would be encountered by fish in the wild since wild animals may potentially move away from the locale of pile driving before the onset of any physiological effects. Moreover, if a wild fish does show an effect from pile driving exposure the results reported here show that recovery may be possible if the fish is not subject to adverse conditions.
